# Altered neocortical oscillations and cellular excitability in an *in vitro Wwox* knockout mouse model of epileptic encephalopathy

**DOI:** 10.1016/j.nbd.2021.105529

**Published:** 2021-12

**Authors:** Vanessa L. Breton, Mark S. Aquilino, Srinivasarao Repudi, Afifa Saleem, Shanthini Mylvaganam, Sara Abu-Swai, Berj L. Bardakjian, Rami I. Aqeilan, Peter L. Carlen

**Affiliations:** a Department of Physiology, Faculty of Medicine, University of Toronto, Toronto, Ontario M5S 1A8, Canada; b Krembil Research Institute, Division of Fundamental Neurobiology, Toronto Western Hospital, Toronto, Ontario M5T 0S8, Canada; c Institute of Biomedical Engineering, University of Toronto, Toronto, Ontario M5S 3G9, Canada; d The Concern Foundation Laboratories, The Lautenberg Center for Immunology and Cancer Research, Immunology and Cancer Research-IMRIC, Hebrew University-Hadassah Medical School, Jerusalem, Israel; e Edward S. Rogers Sr. Department of Electrical and Computer Engineering, University of Toronto, Toronto, Ontario M5S 3G4, Canada; f Department of Medicine (Neurology), University Health Network, Toronto, Ontario M5G 2C4, Canada

**Keywords:** WWOX, Epilepsy, Electrophysiology, NMDA, Coupling

## Abstract

Loss of function mutations of the WW domain-containing oxidoreductase (*WWOX*) gene are associated with severe and fatal drug-resistant pediatric epileptic encephalopathy. Epileptic seizures are typically characterized by neuronal hyperexcitability; however, the specific contribution of WWOX to that hyperexcitability has yet to be investigated. Using a mouse model of neuronal *Wwox*-deletion that exhibit spontaneous seizures, *in vitro* whole-cell and field potential electrophysiological characterization identified spontaneous bursting activity in the neocortex, a marker of the underlying network hyperexcitability. Spectral analysis of the neocortical bursting events highlighted increased phase-amplitude coupling, and a propagation from layer II/III to layer V. These bursts were NMDAR and gap junction dependent. In layer II/III pyramidal neurons, *Wwox* knockout mice demonstrated elevated amplitude of excitatory post-synaptic currents, whereas the frequency and amplitude of inhibitory post-synaptic currents were reduced, as compared to heterozygote and wild-type littermate controls. Furthermore, these neurons were depolarized and demonstrated increased action potential frequency, sag current, and post-inhibitory rebound. These findings suggest WWOX plays an essential role in balancing neocortical excitability and provide insight towards developing therapeutics for those suffering from WWOX disorders.

## Highlights

• Neuronal wwox knockout (s-KO) causes neocortical bursting activity, *in vitro*.
• Bursts are NMDAR and gap-junction dependent, propagating L2/3 to L5.
• Loss of spontaneous inhibition to s-KO superficial neocortical pyramidal neurons.
• Wwox s-KO superficial neocortical pyramidal neurons are hyperexcitable.

## 1 Introduction

Loss of function of WW domain-containing oxidoreductase (*WWOX*) is associated with early infantile epileptic encephalopathy (WOREE/EIEE28), a neurological disorder characterized by severe and uncontrollable epileptic seizures; however, it is currently unknown how a mutation in Wwox results in this epileptic disorder (Abu-Remaileh et al., 2015; Mignot et al., 2015; Valduga et al., 2015; Helbig and Abou Tayoun, 2016; McTague et al., 2016; Tanna and Aqeilan, 2018; Piard et al., 2019). In addition to spontaneous seizure activity, electroencephalography (EEG) recordings from WOREE patients reveal disorganized brain activity, spasms, hypsarrhythmia, slow spikes, and spike-wave discharges (Valduga et al., 2015; Piard et al., 2019), that can be recapitulated in animal models (Suzuki et al., 2009; Mallaret et al., 2014; Tanna and Aqeilan, 2018; Cheng et al., 2020; Repudi et al., 2021). As such, these models provide a useful tool for the study of the mechanisms underlying WOREE, and provide a platform for assessing the function of Wwox in normal brain physiology (Tanna and Aqeilan, 2018). However, this approach necessitates a careful characterization of how a disruption in the WWOX protein manifests in rodents, which as of yet, has not been elucidated.

There are some rodent lines that reproduce the EEG activity seen in WOREE patients. Notably, rats possessing a homozygous frame-shift mutation in *Wwox* show ~10 Hz interictal spiking activity (~10 Hz) in baseline conditions, sound-evoked fast waves during wild-running, and 5-6 Hz spike and wave complexes with bursts of spikes during the clonic phase of sound-induced seizures (Suzuki et al., 2009). Furthermore, spontaneous seizures after postnatal day 12 are also seen in a mouse *Wwox* knockout model (Cheng et al., 2020). Though RNA-seq of neural stem cells from *Wwox* knockout mice show dysregulation of multiple seizure-related genes (Hussain et al., 2019), a characterization of the EEG abnormalities in *Wwox* knockout mice has yet to be done. This characterization is critical to establishing whether *Wwox* knockout mice exhibit similar brain patterns as neonatal epilepsy patients, or possess characteristic biomarkers of epilepsy, such as enhanced low-to-high frequency coupled brain oscillations (Nariai et al., 2011). A recent study showed that neocortical brain slices from neuron-specific Wwox knockouts exhibit spontaneous spike-and-wave complexes, suggesting the neocortex is a driver or these pathological epileptic oscillations, with an underlying neuronal etiology (Repudi et al., 2021).

Reports on brain-specific changes due to Wwox knockout mutations have yet to provide a definitive mechanism for epileptic activity. Cortical hypomyelination is present in a rat model with impaired WWOX function (Tochigi et al., 2019), and in mouse *Wwox* knockout models (Cheng et al., 2020; Repudi et al., 2021); however, hypomyelination alone does not necessarily cause epilepsy (Graciarena et al., 2019; Maas et al., 2020). It is hypothesized that a reduced number of GABAergic interneurons in the hippocampus of *Wwox*-deficient animals may lead to brain hyperexcitability (Hussain et al., 2019). Two previous studies indicate that the mouse neocortex may exhibit similar pathological traits (Iacomino et al., 2020; Repudi et al., 2021). Yet, to the best of our knowledge, there has not yet been any research into the cellular electrophysiological changes occurring in the *Wwox*-deficient mouse cortex, providing a critical gap that needs to be filled. Hence, the first aim of this study is to characterize spike-wave complexes in a neuron-specific Wwox knockout, comparing electrographic activity in the neocortex to the hippocampus. Our second aim is to identify the cellular markers of the WWOX pathology.

## 2 Results

### 2.1 The neocortical network of the neuron-specific WWOX knockout (S-KO) mouse exhibits spontaneous bursting activity, *in vitro*

**Fig. 1:**
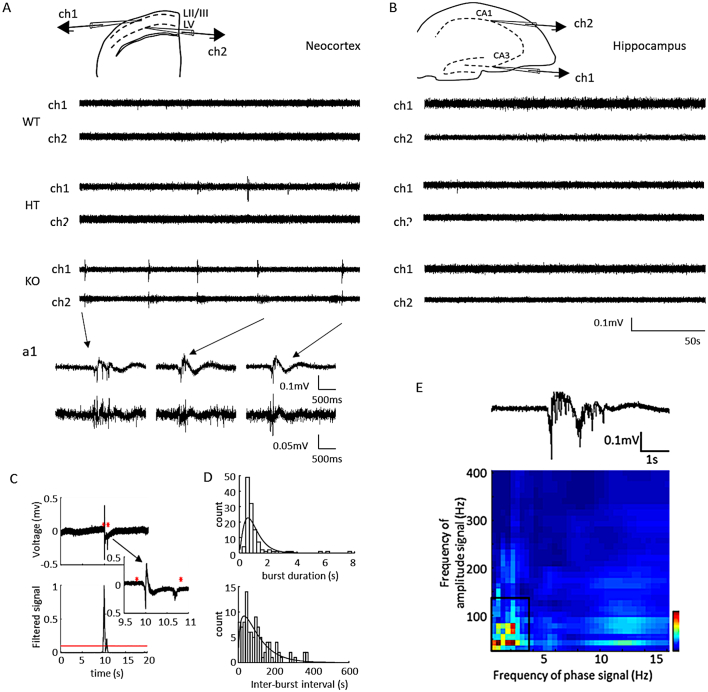
*In vitro* spontaneous bursting activity in the neocortex of Wwox knockout mice. A, Illustration of the placement of field potential recording electrodes in the superficial cortical layer (ch1) and the deep cortical layer (ch2). Field potentials recorded from superficial and deep cortical layers of Cre + wildtype (S-WT), Cre + heterozygote (S-HT) and Cre + knockout (S-KO) littermate mice. a1, Expanded traces of bursting activity present in ch1 and ch2 of the S-KO. Time of bursts indicated by the arrows. B, Illustration of the placement of field potential recording electrodes in the pyramidal cell layer of hippocampal CA3 (ch1) and CA1 (ch2). Field potentials recorded from hippocampal CA1 and CA3 of S-WT, S-HT and S-KO littermates as indicated in A. C, Method used to detect bursting events in the superficial neocortical layer. Stars indicate detected timing of onset and termination of the events. Filtered signal is a convolution of the 4-6 Hz signal with a normalized gaussian (see Methods section for details), highlighting the threshold for event detection. Inset shows expanded trace at times indicated on the horizontal axis. D, Histograms of burst duration and inter-burst interval. The gamma fit distribution of the burst duration has a shape parameter of 2.45 [1.93 3.11] (denoted as: mean [confidence interval of the mean]) for *n* = 119 bursts, 18 slices, 9 animals. The gamma fit distribution of the inter-burst interval has a shape parameter of 1.43 [1.11 1.84] for *n* = 101 inter-burst intervals, 18 slices, 9 animals. Median burst duration, inter-burst interval (converted into frequency (bursts/min) are presented in Supplementary Table S1. E, PAC for a burst showing coupling between delta and gamma frequencies. The peak coupling range is indicated by the black boxes. For summary of peak coupling ranges for all bursts, see Supplementary Table S1.

*Wwox* knockout mice exhibit spontaneous seizures as compared to their heterozygote and wildtype counterparts (Aqeilan et al., 2007; Mallaret et al., 2014; Cheng et al., 2020). Since seizure-generating regions are known to show elevated spontaneous electrographic activity (Nariai et al., 2011; Guirgis et al., 2013; Samiee et al., 2018; Cepeda et al., 2020), we first evaluated the Synapsin-Cre *Wwox* knockout (S-KO) mouse brain for evidence of this epileptic signature. We recorded local field potentials (LFPs) from 13 to 17 day old S-KO, S-HT, and S-WT littermate mouse neocortical and hippocampal slices (Fig. 1). In the neocortical preparations, spontaneous electrographic bursting activity was present in S-KO mice, but not present in S-WT control slices (0 of *n* = 11 slices from 7 S-WT mice; 4 of *n* = 23 slices from 14 S-HT mice; 36 of *n* = 42 slices from 24 S-KO mice) (Fig. 1A; Repudi et al., 2021). In contrast, hippocampal slices exhibited spontaneous activity indistinguishable from controls or heterozygotes (Fig. 1B). The spontaneous activity in the neocortical slices occurred in both layer II/III and layer V; however, it was of larger amplitude in layer II/III as compared to layer V. Using layer II/III activity to measure burst duration and inter-burst interval, the median burst duration was 0.66 [0.53–1.06], whereas the median inter-burst interval was 26.2 s (2.29 [1.1–4.2] bursts/minute).

To investigate how the underlying network communicates during the bursts, we identified if, and at what frequencies, the activity in the superficial neocortex was phase-amplitude cross-frequency coupled (PAC, Fig. 1E, Supplementary Fig. S1). Given that the bursting activity was characterized by these frequency bands, it was also critical to evaluate whether these oscillations were valid PAC signals. First, we inspected the unfiltered signal and observed that high-frequency activity of high amplitude occurred transiently on field bursting activity of varying low frequency waveforms (Fig. 1E, Supplementary Fig. S1A. Then, the power spectrum 10s around each bursting event was computed (Supplementary Fig. S1A, B). Specific peaks were observed in both the low and high frequency bands. These peaks corresponded to coupling between theta (4-6 Hz) and high frequency oscillations (100-400 Hz) (Supplementary Fig. S1C), as well as maximal coupling between delta (0.5-3 Hz) and gamma (30-90 Hz) (Fig. 1E; Supplementary Table S1).

Next, surrogate analysis was used to confirm that the PAC ranges that were identified as coupled were also 3× the standard deviation above the average of the surrogate dataset, demonstrating significant coupling in both ranges. At the maximal frequency range coupled in the theta (Supplementary Fig. S1D) and delta range (Supplementary Fig. S1F, G), we computed the correlation between the I_cfc_ and the wavelet power. If there was significant coupling, then this correlation would either be negatively correlated or not correlated (Jensen et al., 2016). For the theta range, 4/6 showed significant coupling. For the delta range, 8/9 subjects showed significant coupling (Supplementary Fig. S1G). This provided more confidence that the observed delta-to-gamma and theta-to-HFO coupled signals were real.

**Fig. 2:**
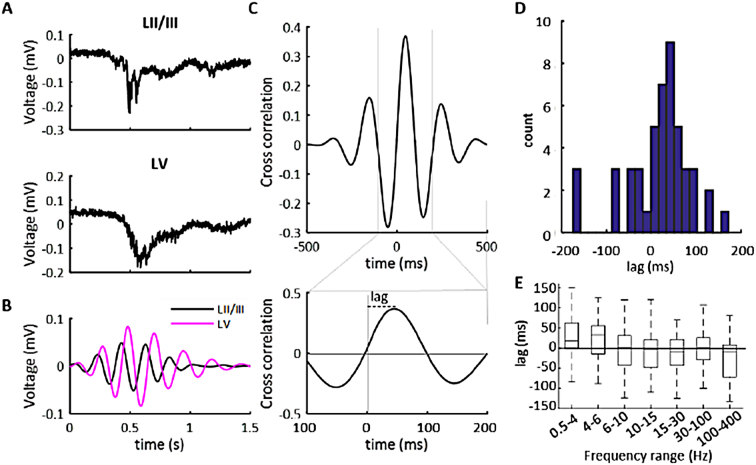
Coupled low frequency oscillations from Wwox knockout mouse neocortical bursting events predominantly propagate from layer II/III to layer V. A, Simultaneous layer V and II/III local field potential recording of bursting activity. B, Layer II/III and layer V recording from A, filtered at 4-6 Hz. C, Cross correlation for the example in A, using the filtered signal (top) and an expanded version of this cross correlation showing computation of lag between the two signals (bottom). D, Histogram of 48 bursts from 17 slices of 6 S-KO animals showing the lag between the layer II/III and layer V signals at 4-6 Hz. E, Boxplot showing lag for varying frequency bands of interest. 0.5-4 Hz shows layer II/III precedes layer V by a median of 18 ms. 4-6 Hz shows layer II/III precedes layer V by a median of 33 ms. 100-400 Hz shows layer V precedes layer II/III by a median of 9.5 ms. Data box is presented in median with 25th and 75th percentiles. Whiskers are the datapoints not considered outliers. Outliers were omitted for clarity.

To identify if this coherent activity represents a propagating waveform, we simultaneously recorded from the superficial and deep cortical layers (Fig. 2A, B) and measured the cross correlation between the two signals at various frequencies (Fig. 2C). Between 0.5 and 6 Hz, layer V lagged behind layer II/III for 32/48 bursts at 0.5-4 Hz and 35/48 burst at 4-6 Hz (median lag at 0.5-4 Hz: 18 [0–62]ms; at 4-6 Hz: 33 [−15–55] ms) (Fig. 2D, E). This suggested that the coherent activity of the low-frequency waveform predominantly propagated from superficial to deep cortical layers. This directionality was not apparent at the higher frequency oscillations.

### 2.2 Gap junctional blockade reduces, whereas glutamatergic blockade abolishes, spontaneous bursting activity

**Fig. 3:**
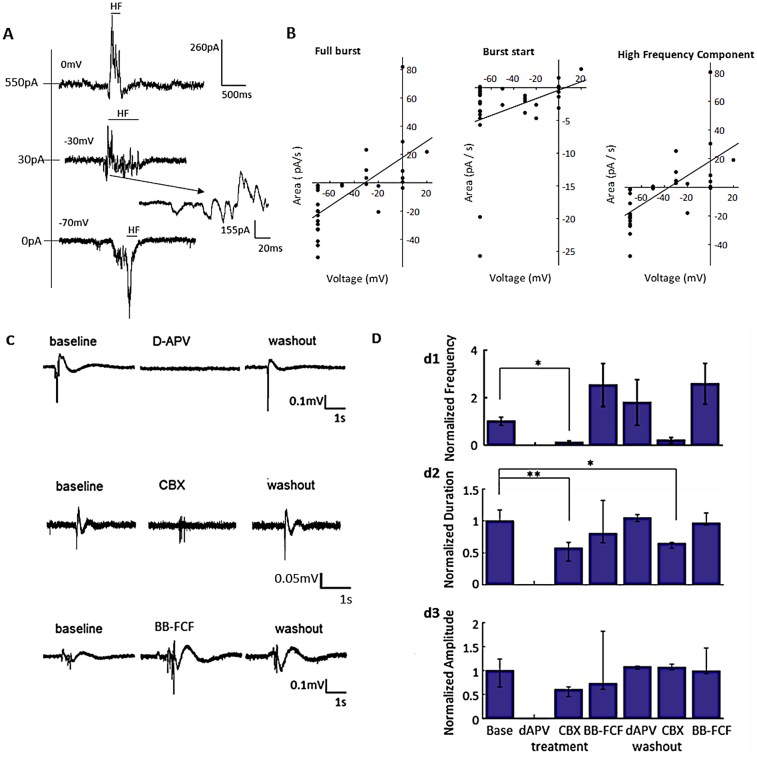
Neocortical bursting activity is initiated by NMDA receptor-dependent excitatory currents. A, Examples of whole cell (WC, bottom) recordings at various holding potentials. Simultaneous LFP and WC recordings are presented in supplementary fig. S2. HF – high frequency component of the bursting event. B, Area under the curve of the voltage clamp recording for: (left) The full burst showing reversal potential of -31 mV, implying a combination of both excitatory and inhibitory currents. (Rho = 0.7499 pval = 1.84e-6 Spearman correlation coefficient; *n* = 30 bursts, 5 animals). (middle) The initial LFP deflection to the onset of the high frequency activity showing a reversal potential of 6 mV, implying dominantly excitatory currents (Rho = 0.3791, *p* = 0.0388 Spearman correlation coefficient). (right) The high frequency component of the bursting activity, with a reversal potential of -36 mV showing a combination of both excitatory and inhibitory currents. The high frequency component of the bursting activity makes up most of the area of the bursting events (rho = 0.8128 pval = 4.8514e-08; Spearman correlation coefficient). C, Examples of the bursts under D-(−)-2-Amino-5-phosphonopentanoic acid (d-APV, 50 μM), carbenoxolone (CBX, 100 μM) and brilliant blue FCF (BB-FCF, 10 μM). D, Normalized frequency, duration and amplitude of bursts under baseline, after pharmacological application of d-APV, CBX or BBfcf, and during washout. (d1: * *p* = 0.008; d2: * *p* = 0.0036, ** *p* = 0.0274; Unpaired *t*-test with Bonferroni's correction; *n* = 51 bursts, 5 slices, 3 animals d-APV; *n* = 36 bursts, 4 slices, 3 animals CBX; *n* = 73 bursts, 6 slices, 3 animals BB-FCF). (For interpretation of the references to colour in this figure legend, the reader is referred to the web version of this article.)

We next sought to investigate how the neocortical bursting activity was generated in the *Wwox* S-KO mouse brains. To approach this, we identified the reversal potential of the events using the technique of whole cell voltage clamp, and we used selective pharmacological antagonists to modulate the events (Fig. 3).

By simultaneously recording intracellularly and extracellularly, we measured the reversal potential of the activity underlying the LFP bursts (Fig. 3A, B). Layer II/III pyramidal neurons were voltage-clamped at different holding potentials (−70 to +20 mV). The total burst duration showed a reversal potential of −31 ± 9.5 mV, assuming a linear model fit (Fig. 3A). Yet, when the time-series was split into an onset window and high frequency component, the onset of the burst showed a reversal potential of 6 ± 11.6 mV (Fig. 3B). This was in contrast to the high frequency component, which showed a reversal potential of −36 ± 9.8 mV. This indicated that excitation, likely glutamatergic, is dominant in these pyramidal neurons at the onset of the bursts (Breton et al., 2020).

Gap junctions are increasingly understood as being involved in the synchronization of neocortical brain oscillations (Mylvaganam et al., 2014), whereas glutamatergic activity appears to dominate the transition to seizure in cortical slice preparations (Huberfeld et al., 2011; Schevon et al., 2012; Shimoda et al., 2020), and are a likely mechanism that initiates these bursts. We therefore investigated the contributions of gap junction and glutamate-receptor mediated neurotransmission to the generation of these bursting events. Upon observing at least three bursting events, indicating consistent bursting activity in the neocortical slice preparations, the slices were perfused with a glutamatergic blocker, d-APV, a non-specific gap junctional blocker, carbenoxolone, CBX, or a selective pannexin blocker, brilliant blue FCF, BB-FCF (Fig. 3C, D). Blocking glutamatergic neurotransmission with d-APV (50 μM) eliminated the spontaneous bursting events, as recorded from an LFP placed in the superficial neocortical layer. Upon washout, the frequency, duration and peak-to-trough amplitude returned to normal. In contrast, CBX (100 μM) decreased the frequency of the events by 87% (*p* = 0.008) and their duration by 18% (*p* = 0.0274, Supplementary Table S1), with no change to their amplitude. This did not return to normal levels after washout. Furthermore, the effects were not recapitulated with BB-FCF (10 μM), indicating that the suppressive effects of CBX were not due to pannexin 1 channel opening. Interestingly, the range of maximal PAC shifted from delta-gamma to delta-HFO upon application of BB-FCF in 10 of 25 bursts evaluated (Supplementary Table S1), suggesting that a subset of these bursts may be influenced by pannexin 1 channels. These data suggest that NMDAR-mediated glutamatergic neurotransmission is a requirement for the generation of these neocortical bursts.

To identify how NMDA receptor-mediated glutamatergic neurotransmission was altered in these neocortical slice preparations, an electrical stimulator was placed in layer V, and the local field potential response to 0.1 ms stimuli was recorded in layer II/III. The response had a delayed negative deflection in the *Wwox* S-KO slices that was not observed in the wildtype controls (Supplementary Fig. S3A). This peaked at 97 ± 31 ms (mean ± standard deviation, *n* = 6 animals) after the stimulus onset. An input-output curve was generated for the amplitude of this delayed response for the wildtypes and knockout mice (Supplementary Fig. S3B), using the ~97 ms peak latency from stimulus onset as a benchmark for evaluating the delay in the wildtypes. The delayed response in the S-KOs was larger than that of the S-WTs at multiple stimulus strengths. This delayed response was NMDA receptor mediated, as addition of d-APV reversibly suppressed the response (Supplementary Fig. S3C). These data support the hypothesis that this neocortical tissue had dysregulated NMDAR-mediated signaling related to the generation of the neocortical bursts.

### 2.3 Postsynaptic, but not presynaptic, glutamatergic changes occur in *Wwox* S-KO neocortical pyramidal neurons

**Fig. 4:**
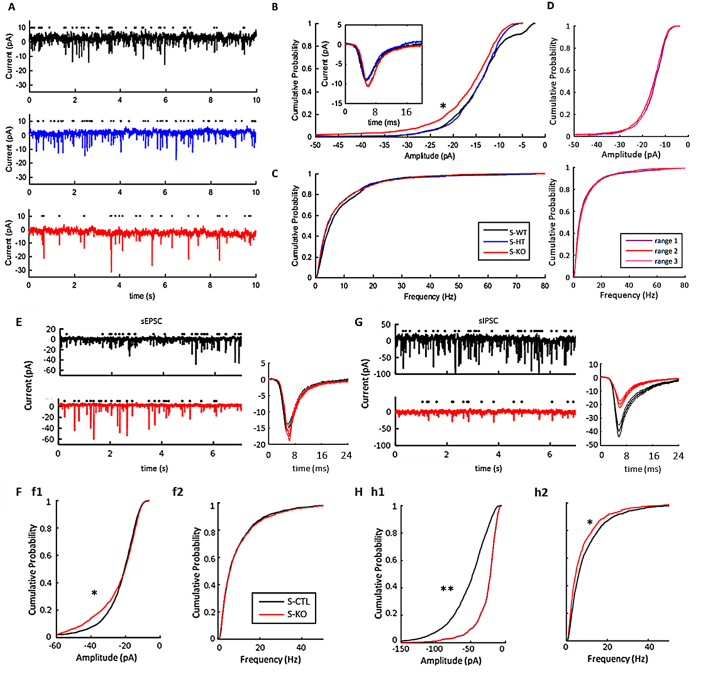
Action-potential independent excitatory currents (mEPSCs) elevated in Wwox knockout mouse pyramidal neurons. A, Examples of mEPSCs for wildtype (S-WT) (black), heterozygote (S-HT) (blue) and knockout (S-KO) (red) obtained by addition of tetrodotoxin, TTX (1 μM), to the perfusate. B, Cumulative probability distribution for the amplitude of the mEPSCs. Inset shows average mEPSCs for S-KO, S-WT and S-HT. (* indicates significance for the following combinations: S-WT vrs S-HT *p* = 1.9831e-04; S-WT vrs S-KO *p* = 4.9906e-07; S-HT vrs S-KO *p* = 1.3306e-06; KS test). C, Cumulative probability distribution for the frequency of the mEPSCs (S-WT vrs S-HT *p* = 0.0018; S-WT vrs S-KO *p* = 2.4054e-04; S-HT vrs S-KO: 0.5963; KS test; S-WT *N* = 10 cells, 4 animals; S-HT N = 10 cells, 5 animals; S-KO N = 10 cells, 5 animals). D, Cumulative probability distributions for amplitude and frequency of the mEPSCs of the S-KO mice at three 50 s time windows within 5 min (range 1, range 2, range 3) (N = 10 cells, 5 animals), demonstrating consistency of currents across time. E, Examples of sEPSCs in the S-WT and S-KO mice. Black dots are the detected events. Right graph shows total mean ± standard error of sEPSCs for all animals. Black indicates control (S-CTL) which is a combination of heterozygotes and wildtypes, red indicates knockout (S-KO). F, Cumulative probability distribution of the amplitudes (f1) (* *p* = 1.5e-5; s-WT vrs s-KO *p* = 3.4659e-23, s-HT vrs s-KO *p* = 5.4976e-26, Kolmogorov–Smirnov (KS) test) and frequencies (f2) (*p* = 0.9805; s-HT vrs s-KO *p* = 0.0315; s-WT vrs sKO *p* = 0.4081; KS test) of the sEPSCs (S-CTLs *n* = 18 cells from 10 animals, S-KOs *n* = 17 cells from 10 animals; s-WT: *N* = 14 cells, 5 animals; s-HET *N* = 17 cells, 8 animals). G, Examples of sIPSCs in the S-WT and S-KO mice. Black dots are the detected events. Right graph shows the total mean ± standard error of sIPSCs for all animals. H, Cumulative probability distribution of the amplitudes (h1) (** *p* = 9.22e-135; sWT vrs sKO *p* = 1.8341e-100; sHT vrs sKO *p* = 2.5249e-69, KS-test) and frequencies (h2) (* *p* = 9.42e-8; sWT vrs sKO *p* = 6.1628e-08; sHT vrs sKO *p* = 4.5624e-05, KS test) of the sIPSCs (S-CTLs *n* = 14 cells from 7 animals; S-KOs *n* = 9 cells from 5 animals; s-WT *N* = 7 cells, 4 animals; s-HET N = 7 cells, 4 animals). (For interpretation of the references to colour in this figure legend, the reader is referred to the web version of this article.)

Increased NMDA receptor signaling can either facilitate presynaptic glutamate release, or enhance postsynaptic processes (Yang and Wang, 2006; Yashiro and Philpot, 2008). Therefore, we studied whether a neuron-specific Wwox deletion results in pre- or postsynaptic changes to glutamatergic currents. We measured action-potential independent miniature excitatory synaptic currents (mEPSCs) by voltage clamping pyramidal neurons at -70 mV and adding TTX (1uM) to the extracellular perfusate (Fig. 4). Cumulative probability plots were generated of the distribution of the amplitude and frequencies of the mEPSCs. There was an increased amplitude of the mEPSCs in the S-KOs as compared to the S-WTs and S-HTs (Fig. 4C). In contrast, there was no change to the frequency of the mEPSCs as compared to S-HTs (Fig. 4C). Furthermore, there were no significant changes in the decay time constant of mEPSCs (S-WT: 5.18 ± 0.78 ms; S-KO: 4.84 ± 1.11 ms; *p* = 0.2982), indicating no overt change in the proportion of the NMDAR:AMPA response (Forsythe and Westbrook, 1988. To confirm the stability of the mEPSCs over time, we subdivided the lengths of the recordings of the KOs into three 50s windows, then re-ran the analysis (Fig. 4D). Consistent frequency and amplitude distributions in TTX were observed, providing confidence of our cumulative probability results. These results were consistent with a postsynaptic mechanistic change to pyramidal neurons.

### 2.4 Evoked neocortical and hippocampal synaptic transmission of the *Wwox* knockout mice

**Fig. 5:**
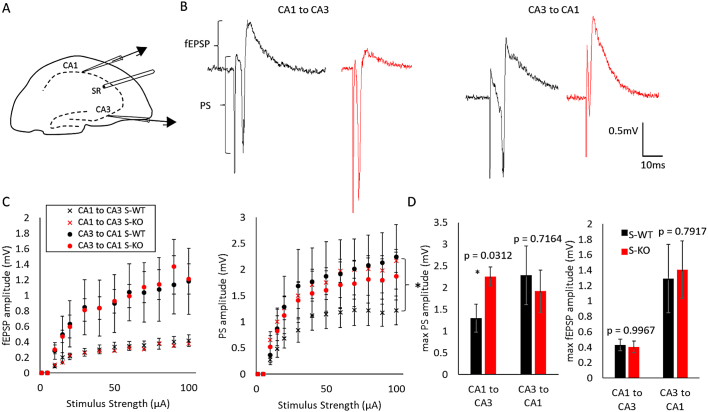
Excitability of Wwox mouse hippocampal circuitry. A, Illustration of placement of local field potential recording electrodes in CA1 and CA3 and stimulating electrode in stratum radiatum (SR). B, Examples of the responses to the two pathways indicated in D at 100 μA stimulus strength. Definitions of field excitatory postsynaptic potential (fEPSP) and population spike (PS) indicated on the diagram. C, fEPSP and PS amplitudes for the two pathways indicated in A (*p < 0.05, Wilcoxon rank sum test for PS amplitude CA1 to CA3 S-KO compared to S-WT stimulus strengths 40-100 μA). D, Maximal PS and fEPSP amplitudes for varying stimulus strengths, normalized to maximal strength for the two pathways indicated in A. No significance (* p indicated as significant, unpaired *t*-test; S-WT *n* = 6 slices, 5 animals; S-KO *n* = 8 slices, 4 animals).

We had previously observed stimulating neocortical layer V and recording layer II/III resulted in large evoked responses (Repudi et al., 2021), indicating that the enhanced spontaneous activity observed in the Wwox S-KO mice is a biomarker of a hyperexcitable neocortical network. Given minimal spontaneous activity was observed in the hippocampus, yet the hippocampus may exhibit a hyperexcitable response to external stimuli, we next asked whether hippocampal networks showed hyperexcitable evoked neurotransmission (Fig. 5). Evoked field potential recordings were made by placing an electrical stimulator in the stratum radiatum, and recording from the CA1 and CA3 (Fig. 5A). In the CA3 to CA1 direction, neither the population spike (PS), nor the field excitatory postsynaptic potential (fEPSP) amplitudes were significantly different compared to the wildtype controls (Fig. 5B, C, D). However, in the CA1 to CA3 direction, there was an elevated PS amplitude in the S-KOs as compared to S-WTs, suggesting that the neocortical hyperexcitability, measured through evoked stimulation, was not observed in the hippocampal circuitry, with one notable exception.

### 2.5 Unbalanced spontaneous excitatory and inhibitory currents in pyramidal neurons of the superficial neocortex

To identify any shift in the balance of excitation and inhibition, sEPSCs and sIPSCs were monitored in layer II/III pyramidal neurons (Fig. 4). There were significantly more large-amplitude sEPSCs in the S-KOs as compared to S-CTLs (a combination of heterozygotes and wildtypes), as indicated by a leftward shift in their cumulative probability distribution (Fig. 4 EA, B, b1; mean amplitude ± standard deviation: S-CTL 23.3 ± 12.0pA; S-KO 24.7 ± 13.6 pA). There was no change in the frequency of sEPSCs (Fig. 4F, f2; S-CTL: 9.9 ± 11.8 Hz; S-KO: 10.1 ± 12.1 Hz; for n's see Fig. 4 legend). Therefore, similar to the mEPSCs, the sEPSCs exhibited an altered amplitude distribution, with an increased probability of higher amplitude events for the S-KOs as compared to controls, but no change in frequency. In contrast, the sIPSCs were reduced in both amplitude and frequency (Fig. 4G, H; mean amplitude: S-CTLs 57.3 ± 31.0pA; S-KOs 27.5 ± 19.4 pA; mean frequency S-CTL 10.3 ± 11.5 Hz; S-KOs 8.4 ± 10.0 Hz; for n's see Fig. 4 legend). These data indicate that the *Wwox* S-KO neocortex favors excitation over inhibition, primarily through an impairment in the amplitude of the inhibitory currents.

### 2.6 Properties of pyramidal neurons and interneurons of the superficial neocortex

**Fig. 6:**
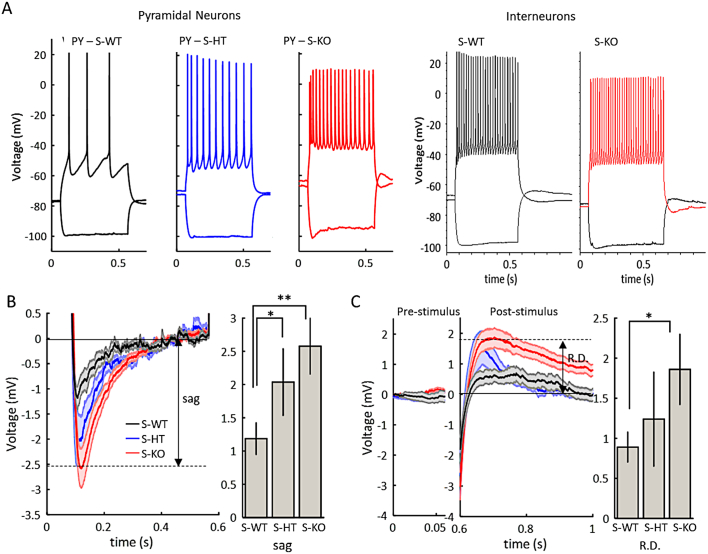
Supra- and sub-threshold patterns of pyramidal neuron and interneurons of the superficial neocortical layers (layers II/III). A, Examples of superficial layer pyramidal neurons (PY) and interneurons (IN) from wildtypes (S-WTs), heterozygotes (S-HTs), and knockouts (KOs). B, Sag voltage for the response to current injection of -200pA (average ± SEM for all animals tested). Arrow on time series indicates definition used for sag voltage. Bar plot shows summary of average sag voltage (* *p* = 0.04761; ** *p* = 0.00322 unpaired t-test). C, Post-inhibitory rebound depolarization (R.D.) for the -200pA current injection. Left curve shows pre-stimulus baseline and middle curve shows post-stimulus R.D.. Arrow on time series indicates definition used for R.D.. Bar plot shows summary of average R.D. (S-WT vrs S-HT *p* = 0.24947; * *p* = 0.00393; unpaired *t*-test). For n's, see Table 1.

Other than the imbalanced synaptic activity, the hyperexcitability of *Wwox* knockouts could also be due to increased pyramidal neuron excitability, decreased interneuron excitability, or both (Fig. 6, Table 1). To test this, we injected 500 ms depolarizing current pulses into neocortical pyramidal neurons and interneurons (Fig. 6A), and plotted the resulting action potential spike frequency as a function of current amplitude (Supplementary Fig. S4). This protocol revealed that there were more action potentials generated in pyramidal neurons of S-KOs as compared to S-WT controls (Supplementary Fig. S4A-a1-b1). Because the S-KO and S-HTs were also more depolarized (Table 1), they may have taken less current to depolarize the membrane to action potential threshold. Therefore, as a second measurement of excitability, we considered the slope of the frequency-current curve, shifting the datasets to one current injection below rheobase (Supplementary Fig. S4A-a2). However, there was no significant difference in the slope of the curve in the S-KOs as compared to the S-WTs and S-HTs (slope – S-WT 0.184 ± 0.044 Hz/pA, S-HT 0.162 ± 0.037 Hz/pA. 0.204 ± 0.070 Hz/pA; S-WT vrs S-HT *p* = 0.1902, S-WT vrs S-KO *p* = 0.3669; unpaired *t*-test; for n's, see Table 1). Furthermore, the interneurons of S-KOs exhibited no significant difference in the number of action potentials generated as compared to controls (Supplementary Fig. S4B), both for shifted and unshifted current injection.

**Table 1:** Electrophysiological features of pyramidal neurons and interneurons of the WWOX S-WTs, S-HTs, S-KOs and S-CTLs (combination of S-WT and S-HT).

	PY – S-WT	PY – S-HT	PY – S-KO	IN- S-WT + S-HT	IN- S-KO
AP half-width (ms)	1.2 ± 0.06	1.3 ± 0.07	1.35 ± 0.07	0.7 ± 0.13	0.55 ± 0.05
AP Threshold (mV)	−43.77 ± 2.47	−40.3 ± 1.40	−41.32 ± 1.09	−40.40 ± 1.76	−42.97 ± 4.77
AP amplitude	76.30 ± 2.2	68.77 ± 2.07*	68.67 ± 1.68*	60.78 ± 3.31	45.03 ± 3.58 *
AHP amplitude	15.14 ± 0.96	17.49 ± 1.33	14.42 ± 0.54	15.42 ± 2.86	16.22 ± 6.37
Input resistance (Mohm)	102.88 ± 11.74	143.26 ± 15.49*	143.03 ± 8.88*	149.97 ± 16.86	118.32 ± 21.22
Resting membrane potential	−79.25 ± 1.99	−75.51 ± 1.81*	−73.62 ± 1.36 *	−67.11 ± 3.81	−71.52 ± 2.28
Tau (ms)	18.55 ± 1.52	18.79 ± 1.77	21.25 ± 1.53	10.58 ± 4.72	10.03 ± 1.82
Sag (at −200 pA)	1.18 ± 0.24	2.04 ± 0.44*	2.57 ± 0.42*	2.03 ± 1.66	2.41 ± 0.55
Post-inhibitory rebound (mV)	0.96 ± 0.19	1.24 ± 0.58	1.86 ± 0.44*	2.67 ± 1.24	2.7 ± 1.07
N's (# cells/# animals)	13/5	15/6	40/15	5/4	6/5

AP – action potential, AHP – afterhyperpolarization. * indicates *p* < 0.05 as compared to S-WT controls. Unpaired t-test for N's indicated in the table. Bold indicates features that are significantly different from controls and that are considered as potential hyperexcitable traits in the S-KOs.

Since these data suggest that the hyperexcitability of the network was not due to action-potential dependent frequency changes, we theorized that subthreshold changes to the cell membrane properties may give rise to the neocortical hyperexcitability. We therefore compared the active and passive membrane properties of neocortical neurons between the S-KOs, S-HTs, and S-WTs (Table 1). We made the following key observations: For pyramidal neurons of S-KOs as compared to S-WTs, the resting membrane potential was depolarized (Fig. 6A), the hyperpolarization activated potassium current was elevated, as evidenced by a larger hyperpolarization in response to a negative current injection, and the post-inhibitory rebound depolarization was increased (Fig. 6). For the interneurons, only the action potential amplitude was diminished in the S-KOs compared to the controls, suggesting the impairment of sIPSCs could be related to reduced action-potential amplitude, but not to other specific active or passive interneuron membrane properties. These data suggest that the control of pyramidal neuron membrane potential is a factor underlying the neocortical network hyperexcitability of *Wwox* S-KO mice.

## 3 Discussion

The mechanism through which *Wwox* S-KO mice get seizures remains elusive. This study (1) characterized neocortical oscillations in *Wwox* S-KO mice, (2) extended the knowledge of the function of WWOX in regulating excitatory and inhibitory networks, and (3) found a series of neuronal changes that coincide with epilepsy-associated brain hyperexcitability. Our results are in support of the critical search for a treatment to stop seizures in newborns with a mutation in WWOX.

### 3.1 Generation of spontaneous activity in slices

We characterized neocortical bursting in *Wwox* neuron-specific conditional knockout mice, *in vitro*. In healthy rodent neocortical slices, this type of activity is not typically detected unless it is induced (Köhling et al., 1998; Avoli et al., 2005). A similar activity can be induced in rodent slices using hyperexcitable media (Avoli and Jefferys, 2016). Yet, spontaneous sharp waves have also been recorded in neocortical slices resected from epileptic patients (Köhling et al., 1998; Tóth et al., 2018). This implies that neuronal deletion of *Wwox* induces a hyperexcitable network, conducive to epileptic seizures. Curiously, these bursts were undetected in the hippocampus, implying a mechanism of hyperexcitability that predominantly affects neocortical circuitry. Given we showed an increased population spike response in the CA3, likely there is hippocampal hyperexcitability, related to axonal conduction, that future studies can address. Also, we cannot discount the possibility of subthreshold excitability changes within hippocampal neurons, that may be undetectable in the physiological range, but may become more pronounced in a hyperexcitable state. Whether this phenomenon indicates cell-type specific influence over excitability (such as through impaired differentiation), or rather a generalized effect (such as hypomyelination) that manifests into bursts only in the cortex, remains to be investigated.

Of note, the spontaneous bursts resulting from WWOX deletion were also observed in heterozygote mice, and manifested similarly as those observed in the knockouts. The presence of these bursts in the heterozygote may be reflective of epileptic network activity; however, there are no clear behavioral seizures in these animals. We cannot discount the possibility that heterozygotes have a form of absence seizures that we are unable to detect visually. However, the more likely explanation is that it reflects a negative correlation between WWOX protein expression and hyperexcitable bursting activity resulting, in the most severe cases, in epileptic activity.

Previously we observed that bursting activity *in vivo* occurs at higher frequency than our *in vitro* model (Repudi et al., 2021). The electrophysiological strategies used to develop our *in vitro* model have been known to lead to neurons being more hyperpolarized, and have a more stable resting membrane potential than neurons recorded *in vivo*, shifting away from the threshold for spontaneous bursting activity (Avoli and Jefferys, 2016; Hartung and Daston, 2009). Furthermore, severing long-distance projections reduces the drive for the propagation of epileptiform activity that is enhanced with thalamocortical connections (Lemieux et al., 2014).

Bursting activity in the WWOX model is primarily propagating from layer II/III to layer V at a velocity of ~11 mm/s. This is similar to the self-sustained neocortical activity that mimics cortical up-states, showing propagation velocity between 10 and 20 mm/s (Sanchez-Vives and McCormick, 2000; Wester and Contreras, 2012). Yet, previous studies show a predominant propagation of neocortical activity from layer V to layer II/III (Sanchez-Vives and McCormick, 2000; Florez et al., 2015). In particular, Sanchez-Vives and McCormick (2000) showed that layer V neurons were relatively more depolarized than layer II/III, increasing their sensitivity to the spread of activity in the network, and therefore discharging first when a slow oscillation arrived. We showed that pyramidal neurons in layer II/III are more depolarized in the *Wwox* knockouts, compared to controls. This implies the directionality of burst spreading may be related to the time it takes to depolarize enough pyramidal neuron membranes to threshold for action potential generation to be visible in the field (Sanchez-Vives and McCormick, 2000).

### 3.2 Phase-amplitude cross frequency coupling in models of pediatric epilepsy

The ranges of cross-frequency coupling observed in *Wwox* S-KO neocortical slices are consistent with other models of epilepsy. Elevated delta-HFO PAC is observed in the spike-wave complexes of rats with a mutation in *Wwox* (Suzuki et al., 2009), mice with MecP2 deficiency (Colic et al., 2014), and in the pilocarpine model of epilepsy (Lévesque et al., 2016). Furthermore, in a cohort of pediatric epilepsy patients, a recent study showed that the amplitude of interictal HFOs (> 80 Hz) was strongly coupled to the phase of 5-10 Hz oscillations (Nariai et al., 2011). Hence, the *Wwox* S-KO spontaneous neocortical bursting, *in vitro,* mimics a key biomarker of pediatric epilepsy, and may continue to give clues as to the underlying changes in cellular dynamics. As HFOs are synchronous with GABAergic potentials before seizure onset, in multiple models of epilepsy (Köhling et al., 2001; Trevelyan et al., 2006; Gnatkovsky et al., 2008; Jefferys et al., 2012), as well as being elevated during the bursts in our model, future studies may look at how the prolongation of the bursts could initiate seizures in *Wwox* S-KO mice. Unlike these previous studies, our study uniquely addresses the neuron-specific KO model of Wwox. Though it is not clear whether the Wwox-null mice exhibit a similar electrophysiological phenotype, the neuron-specificity of this model helps narrow the search for the underlying mechanisms of PAC and its novel relationship with Wwox.

### 3.3 WWOX GABAergic neurotransmission associated with E/I balance

We are the first to demonstrate the changes in the excitatory and inhibitory currents in this *Wwox* S-KO model. Another study showed a loss of GABAergic interneurons in the hippocampus of a ubiquitous *Wwox* S-KO model (Hussain et al., 2019). This is consistent with our observed decreased amplitude and frequency of spontaneous inhibitory currents. As the action potential properties of the interneurons themselves are unchanged in the WWOX S-KO, these findings may indicate a change in pre- or post-synaptic properties unrelated to action potential firing, or reflect a WWOX-dependent decrease in the interneuron population (Hussain et al., 2019) for which we did not account. Our results also agree with the hypothesis of decreased superficial layer GABAergic inhibition underlying the hypersynchronous neocortical network activity (Rhodes et al., 2018). Yet, further studies are likely necessary to identify the specific reason why a Wwox knockout would reduce GABAergic currents. Combining the analysis of epileptiform oscillations with underlying excitatory and inhibitory current networks support how the underlying cellular network changes in this specific pediatric epilepsy model.

### 3.4 NMDA receptor and gap-junctional mediated hypersynchrony as a burst onset trigger

Using both voltage-clamp recordings, and pharmacology, we identified that NMDAR-associated glutamatergic currents initiate neocortical network bursts in *Wwox* S-KO slices. This finding is consistent with a model of Rett syndrome, whose epileptic phenotype manifests as hippocampal bursts of spontaneous glutamatergic activity (*i.e.* regenerative glutamate release (Balakrishnan and Mironov, 2018)) and with many other epileptic models demonstrating that NMDA receptor and gap junction blockade inhibit seizure-like activity (Köhling et al., 2001; Jahromi et al., 2002; Gigout et al., 2006; Heck et al., 2008). Since NMDA signaling contributes to the *Wwox* S-KO epileptic activity, it is of interest to future studies whether long-term potentiation may be altered in this model.

Gap junctions are also said to be involved with the generation of HFOs (> 80 Hz) (Grenier et al., 2003). We showed *Wwox* S-KO pyramidal neurons have increased action potential frequency, as compared to controls. In a previous study, CBX application resulted in increased pyramidal neuron action potential threshold and decreased evoked action potential frequency (Rouach et al., 2003). Hence, gap junctional blockade could be a novel anti-epileptic target for WWOX-related disorders. Yet, CBX may not be specific to gap junctions. First, it could block NMDA receptors (Chepkova et al., 2008), which could partially account for our observations. Second, CBX blocks pannexin channels (Madry et al., 2010), which have the potential to be a novel anti-epileptic target in other epilepsy models (Mylvaganam et al., 2014; Aquilino et al., 2020). However, application of a specific Panx-1 blocker, BB_fcf_, had minimal effect on the network excitability in the *Wwox* S-KO model. Since the recordings were obtained at P13-17, yet these mice die between 3 and 4 postnatal weeks, the chosen age group may mimic a late-stage disorder of WWOX. For this reason, we cannot rule out the possibility that a pannexin 1 blocker could be an anti-epileptic treatment at earlier developmental stages.

### 3.5 Pyramidal neuron excitability through rebound depolarization

Pyramidal neurons of *Wwox* S-KO mice show increased sag current, stronger post-inhibitory rebound, depolarized neuronal membrane potential, and increased evoked spike frequency. The increase in sag current has been correlated directly with increased excitability of layer V pyramidal neurons in human epileptic tissue (Chameh et al., 2019). It has been shown to trigger seizures in rodents through a post-inhibitory rebound excitation (Chang et al., 2018), and is involved in the synchronization of local excitatory networks (Angstadt et al., 2005; Ascoli et al., 2010; Angstadt and Simone, 2014). Moreover, in recordings from hippocampal pyramidal neurons, ZD7288, a hyperpolarization-activated potassium current (Ih) blocker, is shown to cause membrane hyperpolarization and reduced pyramidal neuron excitability through increasing the amount of current required to induce firing (Gasparini and DiFrancesco, 1997). In contrast, in neocortical pyramidal neurons, ZD7288 hyperpolarized the membrane potential, eliminated sag voltage, increased input resistance, but did not alter the properties of the action potentials (Wang et al., 2016). However, it enhanced 4-aminopyridine-induced seizure-like events at high potassium concentrations (Wang et al., 2016), suggesting that the use of an Ih blocker is controversial as an anti-epileptic, and in some cases could make the pathology worse.

### 3.6 Hypomyelination in the *Wwox* knockout model

Our previous work showed neocortical hypomyelination in the neuron-specific *Wwox* knockout model (Repudi et al., 2021), consistent with a global knockout model (Cheng et al., 2020). This could also be a reason why action potentials are reduced in both pyramidal neurons and interneurons in our model. Given that fast-spiking interneurons and oligodendrocyte progenitors (NG2 cells) have coordinated development in the neocortex (Orduz et al., 2015), future studies may look at the relationship between oligodendrocyte-specific loss of WWOX and the activity of selective neocortical interneuron subtypes. Furthermore, it is also possible that ephaptic coupling may be strengthened through a lack of myelin (Hartline, 2008). These ephaptic connections are particularly relevant for entraining action potentials and low frequency oscillations (Anastassiou et al., 2011), both of which we observed as elevated in the hyperexcitable *Wwox* S-KO brain tissue. Hence, future studies may also look at ephaptic coupling as a mechanism which drives the pathological oscillations in the *Wwox* S-KO mice.

## 4 Conclusions

Key electrophysiological results show the complexity of the epileptic disorder of the *Wwox* S-KO model. Main pathophysiological changes in the *Wwox* mouse neocortex are the following: 1) decreased spontaneous inhibition 2) increased gap junctional and NMDAR mediated activity, 3) pyramidal neuron membrane depolarization, and 4) increased post-inhibitory rebound excitation. This work is a critical step towards identifying novel anti-epileptic therapies for pediatric patients with this devastating and fatal pediatric epileptic encephalopathy due to *WWOX* gene mutations.

## 5 Methods

### 5.1 Animals

Mice carrying two *loxp* sites (*Wwox*^*flox/flox*^) flanking Exon 1 of the *Wwox* genomic locus was previously documented (Abdeen et al., 2013). Conditional ablation of *Wwox* expression in neurons is achieved by crossing male mice possessing one floxed allele of *Wwox* and Synapsin I Cre (JAX stock #003966) with mice carrying single or both floxed allele of *Wwox*. Mice were separated into three experimental groups based on genetic profiles: homozygous knockout (S-KO, Wwox^fl/fl^ and Cre+), heterozygotes (S-HT, Wwox^fl/+^ and Cre+), and controls (S-WT, Wwox ^+/+^ and Cre+). Where no significant difference exists between S-HT and S-WT groups, they are occasionally represented as a clustered group (S-CTL) for clarity. Both male and female mice aged P13 to P17 were humanely sacrificed for these experiments in accordance with the guidelines outlined by the Canadian Council of Animal Care (CCAC). All surgical procedures were approved and done in accordance with the guidelines of the Animal Care Committee of the University Health Network.

Genotypes were identified by PCR using the following oligonucleotide primers: wildtype allele: Forward: 5’-AGGGACGGCTGGGTGTACTA-3′; Reverse: 5’-CAACCTACTAGCCTCTCCAC-3′; (540 pb) floxed allele: Forward: 5’-AGGGACGGCTGGGTGTACTA-3′, Reverse: 5’-ACCAAAGAACGGAGCCGGTT-3′ (800 bp); synapsin Cre: Forward – 5’-CTCAGCGCTGCCTCAGTCT-3′; Reverse: 5’-GCATCGACCGGTAATGCA-3′ (~300 bp).

### 5.2 *In vitro* slice preparation

Mice were anesthetized with pentobarbital (50-mg/kg). Depth of anesthesia was tested using the pedal reflex. Once the mice were deeply anesthetized, they were swiftly decapitated, and the brain was removed. The cerebellum and olfactory bulbs were removed, and the remainder of the tissue was placed caudal-side down onto a platform in a solution of ice-cold sucrose containing (in mM): 248 sucrose, 26 NaHCO3, 10 glucose, 2 KCl, 3 MgSO4-7H2O, 1.25NaH2PO4, 1CaCl2-2H2O. The neocortex and hippocampus were sectioned coronally 400–500 um thick (0.6 mm/s speed, 1 mm amplitude) using a Leica 1200 vibratome. After this, slices were incubated in artificial cerebral spinal fluid (ACSF) containing (in mM): 123 NaCl, 25 NaHCO3, 10 glucose, 3.5 KCl, 1.3 MgSO4-7H2O, 1.2 NaH2PO4, 1.5 CaCl2-2H2O. Slices were kept at 35^o^ for 30 min, after which they were removed to room temperature for at least 60 min prior to experimentation at 35^o^.

### 5.3 *In vitro* slice electrophysiology

Local field potential (LFP) glass electrodes (1.5 mm, World Precision Instruments) containing the ACSF were pulled using a vertical puller (Narishige, Japan PP-83) and positioned in neocortical layers II and III, V, or in the CA1 and CA3 regions of the hippocampus. An Olympus BX51 microscope (OLY-150IR camera–video monitor unit) was used as guidance for proper electrode placement. To assess network excitability, electrical stimulation was performed using a bipolar concentric tungsten electrode. Current pulses of 0.1 ms duration with varying strengths were applied every 30s using a GRASS S88 stimulator connected to a photoelectric stimulus isolation unit. Evoked stimuli were first repeated at a constant strength for at least 5 min to achieve inter-response stability. Then, evoked responses were done in triplicate to ensure reproducibility of results, then an average of the evoked response was reported.

An Olympus BX51 microscope (OLY-150IR camera–video monitor unit) was used with an infrared filter to visualize the neurons. The whole cell recording electrodes (3-5 MΩ resistance) contained a solution of (in mM): 135 K-Gluconate, 1 MgCl2, 10NaCl, 2 Na2ATP, 0.3 NaGTP-Tris, 10 NaHEPES, 0.5 EGTA, 0.0001 CaCl2, pH 7.2–7.3, 270–290 mOsm. For recordings of sIPSCs, a high-chloride intracellular solution was used, in order to enhance the chemical gradient of chloride. This solution is composed of, in mM: 140 KCl, 1 CaCl2, 1 MgCl2, 10 EGTA, 10 HEPES, 3 Mg-ATP, 0.3 Na2GTP, pH 7.2, 295 mOsm. Signal acquisition and storage were performed using an amplifier (Muliclamp 700B), a digitizer (Digidata 1322A) and PClamp software (version 10.2; Axon Instruments/Molecular Devices Corporation). Whole cell recordings were done in either current clamp or voltage clamp mode. Putative pyramidal neurons were identified based on their appearance under the infrared filter and their electrophysiological features by performing a series of hyperpolarizing and depolarizing current pulses (500 ms duration) through the whole cell recording electrode. We did not correct for liquid junction potential since all results central to the hypothesis were measured against a change from wildtype littermate controls. To assure that only healthy neurons were used, only those that were not spiking spontaneously were recorded. Intrinsic electrophysiological properties were recorded and access resistance was monitored throughout. Cells were not used if the access resistance was >40 MΩ and if they had an unstable membrane potential.

Spontaneous EPSCs and IPSCs were isolated by voltage-clamping pyramidal neurons at -70 mV in K-Gluconate or KCl intracellular solution respectively.

### 5.4 Pharmacology

Initial stock solutions of tetrodotoxin (TTX), 2-amino-5-phosphonopentanoic acid (d-APV), carbenoxolone (CBX) and brilliant blue FCF (BB-FCF), dissolved in double-distilled water, were further individually diluted in ACSF for a final concentration of 1 μM TTX, 50 μM d-APV, 100 μM CBX, and 10 μM BB-FCF.

## 6 Signal processing

### 6.1 Burst detection

As a consistent means to identify burst duration and inter-burst interval, the signal was first band-pass filtered at 4-6 Hz using a 10,000 order finite impulse response (FIR) filter run in the forward and reverse direction (filtfilt.m, Matlab). Then, the square of the filtered data was convolved with a Gaussian kernel (200-point aperture) (Colic et al., 2014). The upper envelope of that signal was obtained (envelope.m, Matlab) and a threshold (10% of maximum power) was used to mark the onset and termination of the bursting events (Fig. 1C). These time-points were confirmed visually to compute the burst duration and inter-burst interval (Fig. 1D). Burst duration and inter-burst interval were plotted using the histfit.m function in Matlab, fitting the data to a gamma distribution (Breton et al., 2020).

### 6.2 Time-frequency spectral analysis

The time-frequency spectrograms (Supplementary Fig. S1A) were generated by convolution of the LFP signal with complex Morlet wavelet with bandwidth of 5-Hz and 0.8125-Hz center frequency, then z-score normalized. The average and standard error z-score normalized to a quiescent baseline period prior to burst onset is presented in Supplementary Fig. S1B.

The phase amplitude cross frequency coupling (PAC) analysis was computed as previously described (Breton et al., 2019). The instantaneous phase of the low frequency oscillations (1-12 Hz) and amplitude of the high frequency oscillations (30-250 Hz) were computed by convolution of the filtered LFP signal with the complex Morlet wavelet. To avoid edge effects, a 5 s window on either side of the signal of interest was kept during the computation of the continuous wavelet transform (cwt), and then subsequently removed for calculation of the cross-frequency coupling indices (I_CFC_). Then the I_CFC_ was estimated on a 10s window centered at the midpoint of each burst. The instantaneous phase signal was divided into 20^o^ equal intervals. In each phase interval, the amplitude, *A*_*high*_, was averaged then normalized by the sum of the average amplitudes (Tort et al., 2010). The Kullback-Leibler (KL) distance was computed between the normalized amplitude, A_norm_, and a uniform distribution. Then, the KL distance was normalized to an interval between 0 and 1. This normalized KL distance was the phase amplitude cross frequency coupling index (I_cfc_) (also known as modulation index) for each data point on the image (Supplementary Fig. S1C,E).

### 6.3 I_CFC_ control analysis

To identify significant ranges of PAC, surrogate data was generated by converting the original time series into a complex series using the cwt. The phase was then random block-point shuffled with blocks of 1% of the sampling frequency (10 data points, randblock.m). Then the I_CFC_ was recomputed. The amplitude coefficients of the high frequency cwt were used, with the surrogates, to compute the I_CFC_. This was repeated 100 times. The original PAC was z-score normalized to the mean and standard deviation of the distribution of the surrogate values. Ranges considered significant were those whose distributions were above 3× the standard deviation of the mean of the surrogate dataset. This test for significance provided the boundaries of significant PAC for further control analysis. As a second measurement of significance for the PAC, the cwt power was plotted against the maximum I_CFC_ value as a scatter plot (Supplementary Fig. S1D,F). If the line of best fit is negative or if the R value (rho) is negative, then the dataset more likely contains true PAC (Jensen et al., 2016).

### 6.4 Cross-correlation analysis

Cross-correlation between simultaneously recorded layer II/III and layer V LFP signals was performed in Matlab. First, the data were decimated to 1000 Hz. Then the dataset was filtered to selective frequencies using a bandpass Hamming window filter (5000 order, fir1). The lag was computed using the xcorr.m function.

### 6.5 Spontaneous current analysis

The area under the curve of the currents was identified using the Trapz.m in Matlab. The signal was decimated to 1000-Hz. A bandpass filter was designed using the kaiserwin method and the following parameters: stopbandfrequency1 (5-Hz), PassbandFrequency1 (15-Hz), PassbandFrequency2 (150-Hz), StopbandFrequency2 (200-Hz), StopbandAttenuation1 (60-Hz), PassbandRipple (1-Hz), StopbandAttenuation2 (40). The threshold detection was set at 4× the standard deviation of the filtered signal. The window size used for detection was 100 ms, with a baseline window of 10 ms. Amplitude and decay time constant were adjusted to ensure the automatically detected events matched our visual identification. Cumulative probability plots of the miniature and spontaneous excitatory post-synaptic current (mEPSC and sEPSC, respectively), and spontaneous inhibitory post-synaptic current (sIPSC) amplitude and frequency were generated using ecdf.m function in Matlab, which computes the Kaplan-Meier estimate of the cumulative distribution function of the input. This shows the probability that the data is less than or equal to the values shown on the horizontal axis. A change in the frequency may indicate that a presynaptic mechanism of action is likely due to the release probability of presynaptic neurotransmitter (Fatt and Katz, 1952). In contrast, the amplitude of a mEPSC is dependent on several different factors, such as postsynaptic glutamate receptor sensitivity, the amount of glutamate released, and the ionic driving force underlying the postsynaptic current (Scanziani et al., 1992).

### 6.6 Electrophysiological Properties of pyramidal neurons and interneurons

Clampfit (Clampex 10.2.0.12) was used to compute the specific electrophysiological properties of the layer II/III neurons. Electrophysiological features were obtained by performing a series of hyperpolarizing and depolarizing current pulses (500 ms duration) through the whole cell recording electrode. Action potential (AP) and afterhyperpolarization (AHP) analysis was done on the first spike induced at rheobase. Action potential frequency was calculated by counting the number of action potentials at each depolarizing current pulse using a custom Matlab script.

For the AP characterization: the AP threshold was measured at the point of steepest voltage change, amplitude was measured from the threshold to the peak of the AP. Half-width was measured at the position half-way between the threshold and the peak of the action potential. AHP amplitude was measured from the threshold to the most hyperpolarized value immediately after the AP.

Other membrane properties were defined through the following calculations: Resting membrane potential (RMP) was identified as the average 50 ms prior to the first intracellular stimulus. Input resistance was measured by the slope of the computed I-V plot. The time constant was measured by applying the first 50-60 ms of a small hyperpolarizing stimulus to an exponential, standard fit. The sag voltage was measured by subtracting the most negative membrane deflection from its return to steady state in response to a hyperpolarizing pulse of -200pA. Post-inhibitory rebound depolarization was measured by the most positive membrane deflection immediately following a -200pA stimulus to the average of the first 50 ms prior to the stimulus.

Putative pyramidal neurons were identified based on their appearance under the infrared filter and their electrophysiological features. They were later confirmed based on comparison of the spike-half width, input resistance, and membrane time constant.

### 6.7 Experimental design and statistical analysis

Statistical design for the experiments can be found in the results section describing the figures. Sample size represents at least the minimum number of samples needed to establish significance. Littermate wildtype and heterozygote controls were used for this study. Statistical analyses were performed using Matlab. An unpaired *t*-test was used when there were two groups and a normal distribution, and Wilcoxon rank-sum test when there were two groups and a non-normal distribution. A Bonferroni post-hoc correction was applied in each case for tests of multiple comparisons. A Pearson correlation coefficient was applied to measure the linear correlation between the duration of the sub-intervals of the SLE state S2 and that of the duration and intensity of the SLE state S2 (0 → no correlation, 0.1–0.3 → weak correlation, 0.31–0.69 → moderate correlation, 0.7–1 → strong correlation).

## Declaration of Competing Interest

The authors declare no conflict of interest.

## Appendix A Supplementary data

Supplementary material Image 1
