# Supplementary material for: Altered neocortical oscillations and cellular excitability in an in vitro Wwox knockout mouse model of epileptic encephalopathy
Source: Neurobiol Dis. 2021 Dec;160:105529. doi: 10.1016/j.nbd.2021.105529 (PMC8609180; doi:10.1016/j.nbd.2021.105529)
Supplement: Supplementary file 1 — Supplementary material [file mmc1.docx]

Supplementary Data for

**Altered neocortical oscillations and cellular excitability in a Wwox knockout mouse model of epileptic encephalopathy in vitro**

Breton VL*^1,2^, Aquilino MS*^2,3^, Repudi S^4^, Saleem A^2,3^, Mylvaganam S^2^, Sara Abu-Swai^4^, Bardakjian BL^3,5^, Aqeilan RI^4^, Carlen PL^1,2,3,6^

^*^ These authors have contributed equally, and share first authorship

^1^ Department of Physiology, Faculty of Medicine, University of Toronto, Toronto, Ontario, Canada M5S 1A8
^2^ Krembil Research Institute, Division of Fundamental Neurobiology, Toronto Western Hospital, Toronto, Ontario, Canada M5T 0S8
^3^ Institute of Biomedical Engineering, University of Toronto, Toronto, Ontario, Canada M5S 3G9,
^4^ The Concern Foundation Laboratories, The Lautenberg Center for Immunology and Cancer Research, Immunology and Cancer Research-IMRIC, Hebrew University-Hadassah Medical School, Jerusalem, Israel;
^5^ Edward S. Rogers Sr. Department of Electrical and Computer Engineering, University of Toronto, Toronto, Ontario, Canada M5S 3G4
^6^ Department of Medicine (Neurology), University Health Network, Toronto, Ontario, Canada M5G 2C4

|  | Baseline | CBX | BB-FCF |
| --- | --- | --- | --- |
| Frequency (bursts/min) | .84 [.57-.1.53]; min 0.36, max 3.89 | 0.03 [0.03-0.05]; min 0 max, .15 | .78 [.44-.1.09]; min .12, max 25.68 |
| Duration (s) median [25^th^, 75^th^ percentiles] | 0.66 [0.53 1.06]; min 0.26; max 6.10 | 0.54 [0.52 0.73]; min 0.50 max 1.61 | 0.53 [0.51 1.46]; min 0.45; max 2.84 |
| Amplitude (mV) | 0.36 [0.30 0.53]; min 0.12, max 1.01 | 0.53 [0.32 0.74]; min 0.11 max 0.91 | 0.38 [0.19 0.49]; min 0.11 max 0.72 |
| Max PAC value | 0.024 [0.014 0.039]; min 0.0028, max 0.1637 | 0.0094 [0.0015 0.0388]; min 0.0013, max 0.0645 | 0.074 [0.043 0.091]; min 0.0051, max 0.18 |
| Max PAC low frequency range | 0.5-1.5 [0.5-3]; min 0.5, max 14 | 0.5 – 2.5 [0.5 4.25]; min 0.5, max 6 | 0.5 – 2.5 [0.5 14]; min 0.5, max 16 |
| Max PAC high frequency range | 30-80 [30 – 90]; min 30, max 400 | 30-70 [30 - 80]; min 30, max 120 | 30-122.5 [30 - 365]; min 30, max 400 |
| N’s (bursts/slices/animals) | 119/18/ 9 | 13/6/3 | 25/6/3 |

**Supplementary table S1.** Summarized properties of the neocortical bursts. PAC – phase amplitude cross frequency coupling, CBX – carbenoxolone, BB-FCF – brilliant blue FCF. This table summarizes the findings of Figures 1-4.


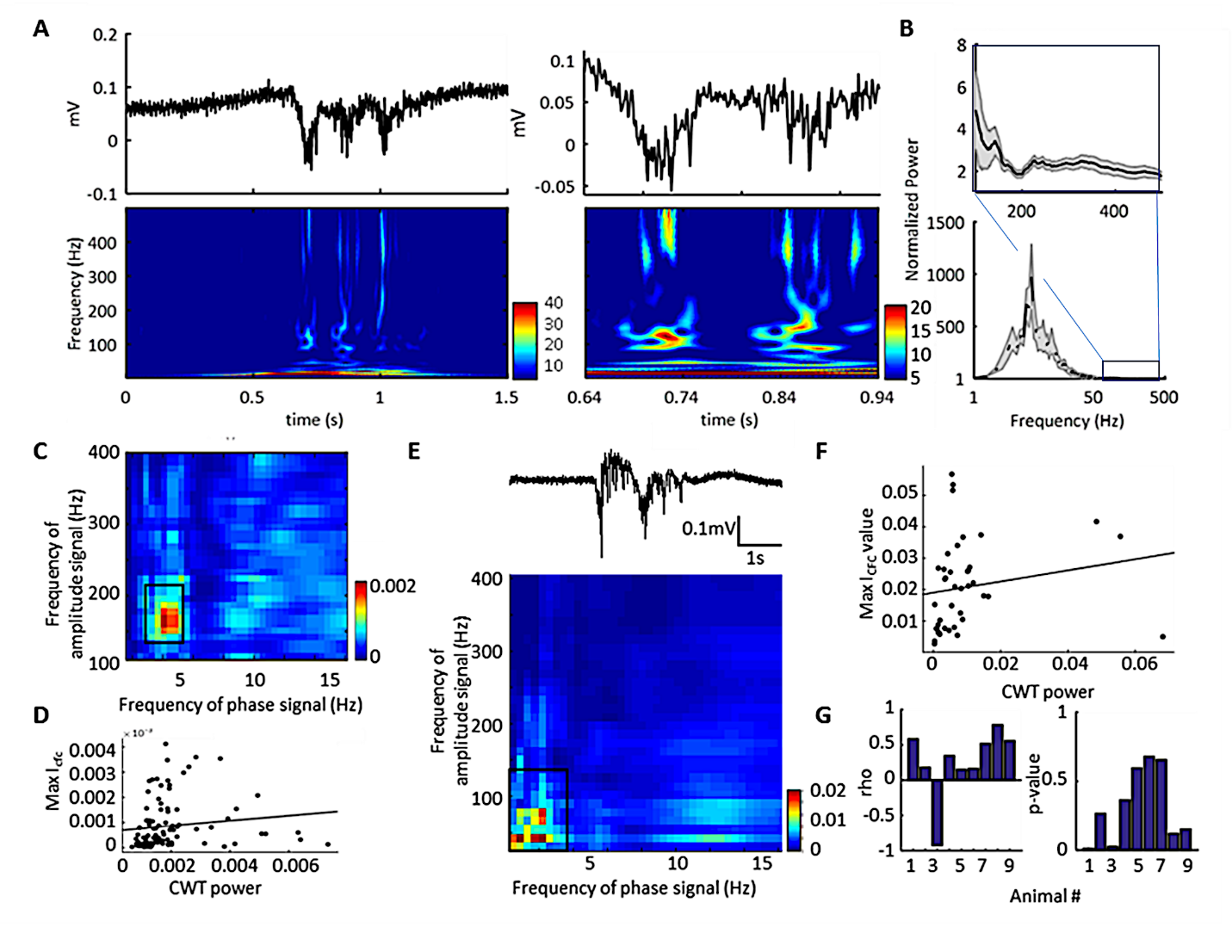


**Supplementary Figure S1:** Time-frequency spectral analysis of neocortical bursting activity of Wwox knockout mice showing elevated coupling between low and high frequency oscillations. A, Local field potential recording of spontaneous bursting activity with a z-score normalized time-frequency spectrogram. Right-hand trace is an expanded view of the left trace with scale bar adjusted to demonstrate the high frequency activity which is coupled to low frequency intervals. B, Power spectral analysis of high (top) and low (bottom) frequency power spectra z-score normalized of a 10s trace surrounding the middle of the burst event. Data presented in mean +/- standard error. C, Phase amplitude cross frequency coupling (PAC) for the trace in A showing peak coupling in the 4-6Hz and 140-200Hz frequency ranges. D, Scatter plot of 102 bursts from 11 slices of 6 S-KO animals showing no significant correlation between the power of the low frequency phase signal where there is maximal coupling and the cross frequency coupling index (Icfc) obtained at the maximal coupling strength (rho = 0.1283, p = 0.1988; Pearson Correlation Coefficient). E, PAC for a second example burst showing coupling between delta and gamma frequencies. The peak coupling range is indicated by the black boxes. For summary of peak coupling ranges for all bursts, see supplementary table S1. F, Scatter plot of 41 bursts from 5 slice of 1 S-KO animal showing no significant correlation between the power of the low frequency phase signal where there is maximal coupling and the Icfc obtained at the maximal coupling strength (rho = -0.1201, p = 0.5674; Pearson Correlation Coefficient). G, Rho and p values for 126 bursts in 18 slices of 9 animals showing no significant correlation or negative correlation in 8/9 of the subjects.


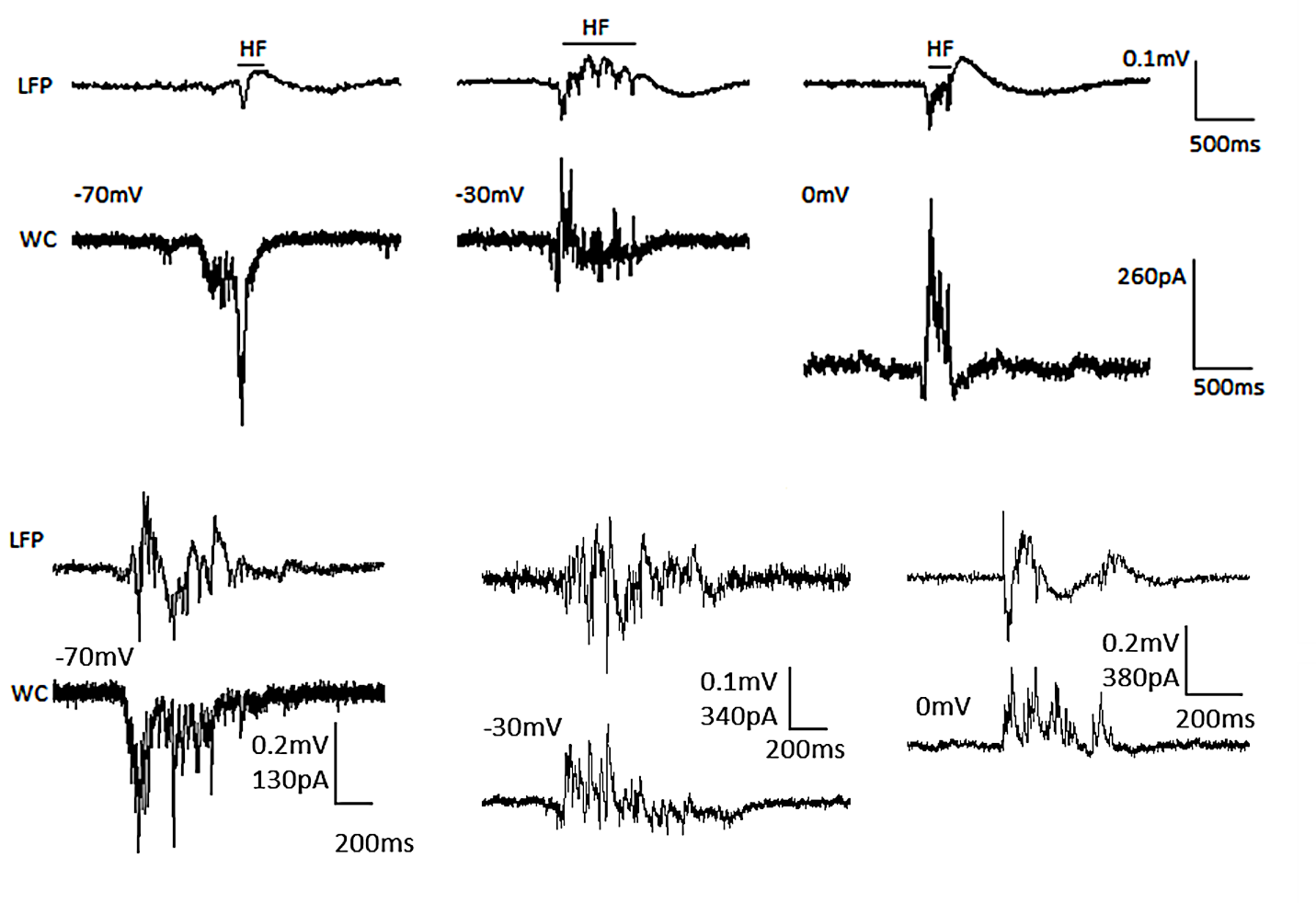


**Supplementary Figure S2:** Examples of simultaneous local field potential (LFP, top) and whole cell (WC, bottom) recordings at various holding potentials.


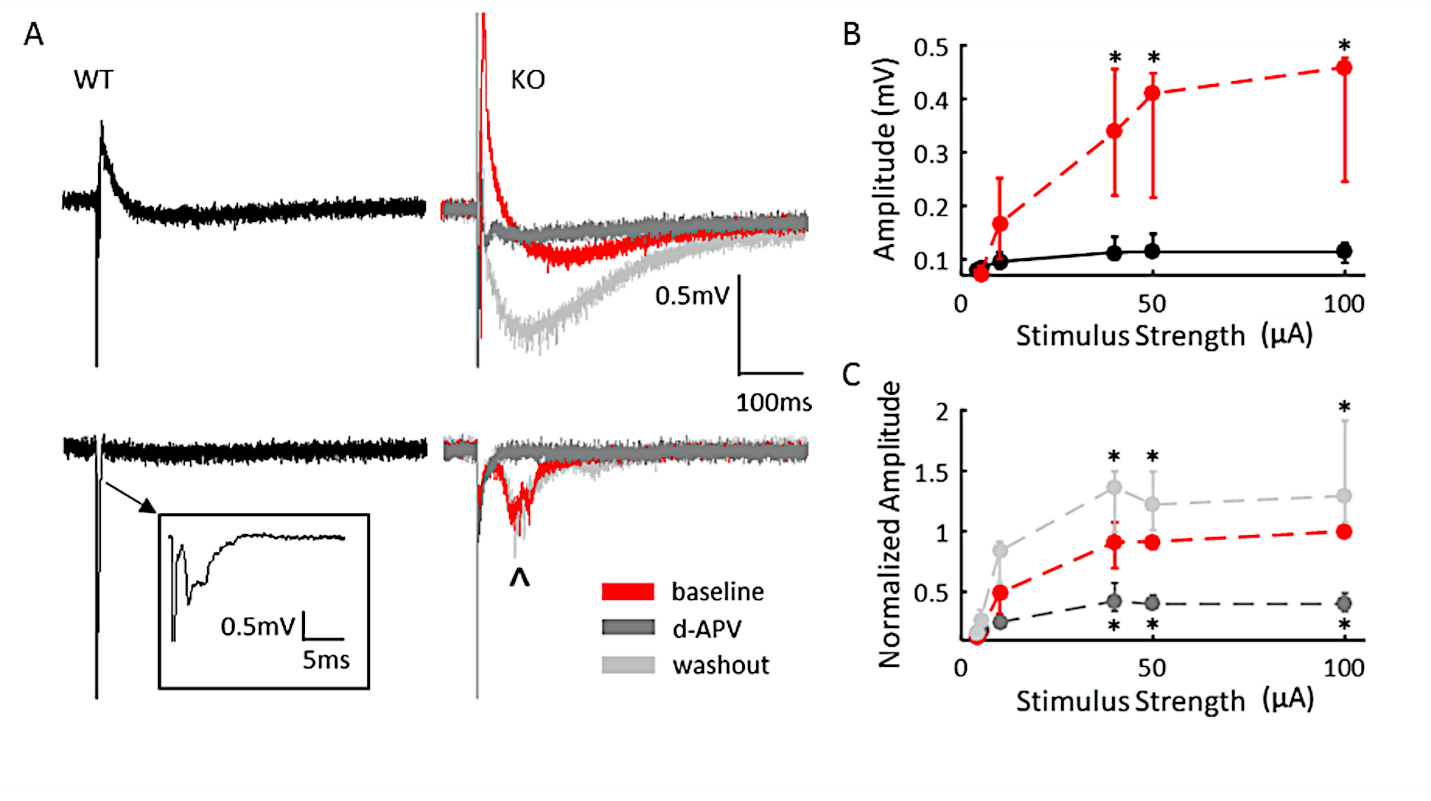


**Supplementary figure S3:** NMDA-receptor blockade suppresses delayed field-evoked activity between neocortical layer V and layer II/III. A, Examples of field evoked responses in layer II/III in the wildtype controls (S-WT) as compared to the knockouts (S-KO) at various positions in the deep neocortical layer relative to the recording electrode (top – along the same cortical column, bottom – in the adjacent cortical column). The caret ‘^’ symbol shows the peak of the delayed evoked response used to calculate the amplitude. B, Amplitude of the delayed evoked response for the S-WT as compared to the S-KO mice (‘*’ indicate significant points as compared to baseline stimulation. from left to right, p = 0.5713, 0.4207, 0.1554, 0.011, 0.0092, 0.0038, unpaired t-test; n = 6 slices, 3 animals S-KO, n = 7 slices, 4 animals S-WT). C, Amplitude of the delayed evoked response at baseline, during d-APV treatment, and after washout. Data was normalized to a stimulus strength of 100μA. (p values relative to baseline for d-APV treatment are (left to right) 0.4798, 0.6458, 0.1692, 0.0229, 0.0103, 0.0006; p-values relative to baseline for washout (left to right) are 0.9425, 0.2524, 0.1975, 0.0492, 0.0372, 0.0499. unpaired t-test n = 6 slices, 3 animals).


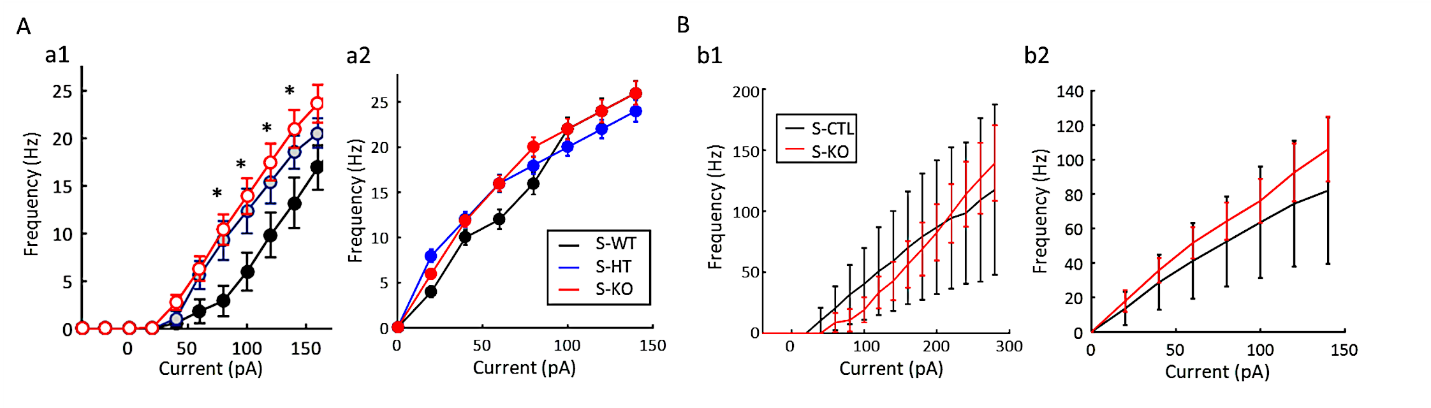


**Supplementary figure S4.**A, a1, Frequency of action potentials relative to raw injected current strength for increasing stimulus amplitudes for pyramidal neurons (Significance shown for knockout compared to wildtype; * (from left to right) p = 0.012443 unpaired t-test; p = 0.023729 unpaired t-test, p = 0.034794 rank sum test, p = 0.04364; rank sum test). Data presented in mean +/- standard error. a2, Frequency of action potentials where zero injected current represents 20pA below rheobase for each pyramidal neuron (p > 0.05 for all combinations tested, unpaired t-test). B, b1, Frequency of action potentials relative to injected current strength for increasing stimulus amplitudes for interneurons (p > 0.05 for all combinations tested, unpaired t-test). b2, Frequency of action potentials where zero injected current represents 20pA below rheobase for each interneuron (p > 0.05 for all combinations tested, unpaired t-test). For n’s see table 1.
